# The Hygiene of the Mouth

**Published:** 1901-07-15

**Authors:** Byron L. Kesler

**Affiliations:** Salt Lake City, Utah


### THE HYGIENE OF THE MOUTH.

BY BYRON L. KESLER, D.D.S., SALT LAKE CITY, UTAH.

A distinguished contributor to medical literature makes the statement that the science of medicine, during the century just closed, achieved its greatest triumphs in "Preventive Medicine."[1] The profession, through local, State and national boards of health, has advised proper regulations and sanitary measures for preventing the dissemination of those epidemic diseases which at times have threatened to depopulate the globe.

The term *Prophylaxis* is derived from the Greek word meaning "I defend," or "to guard against." The *prevention of disease* stands to-day in the front rank of medical practice. To the practitioner of general medicine it is one of his most effective weapons against the common enemy, infection. It is of even

1. "Pseudo Medicine," lecture by Prof. Vaughan, New York and University of Michigan Medical Societies, 1900.

greater importance to the "common people," as an efficient means of protection against the scourges, plagues and contagions which afflict the people of those countries where ignorance, filth, superstition and uncleanliness abound.

In this age of rapid and quick communication, with its increased facilities for transportation, the dissemination of contagious diseases should be accelerated; but contemporaneously our knowledge of more perfect methods for the prevention and control of disease is correspondingly increased, thus allaying fears for the future and inspiring confidence in the investigations of science and the shield of protection which it affords.

The statement is made by Professor Vaughan, in speaking of *mental hygiene*, that the *mind* must be studied from the view point of the materialist; that the mind can not be considered as an entity, dissociated from the brain, as the operations of the mind are merely the physiological phenomena of brain function;[2] that a sound mind is dependent upon a sound body, and a sound body is dependent upon a perfect *digestion*, genius, even, being unable to compensate for the ill-temper produced by a faulty digestion. Logically may we not, therefore, go a step further and say that a perfect digestion is largely dependent upon, or is greatly influenced by a thorough *mastication* of the food, and that mastication is dependent upon a good set of *teeth*? Therefore the necessity for dental sanitary science, or dental surgery.

As it is practically impossible to be a good Christian when suffering from indigestion, regardless of theory and pretensions, mastication certainly, and salvation

2. "Mental Hygiene," lecture by Prof. Vaughan, March 29, 1901.

probably, in a great measure may depend upon the skill and knowledge of one's dentist.

If sanitary science is important in the practice of general medicine, it is of even greater importance in oral hygiene. The old but true axiom, that "cleanliness is next to godliness," may be applied to the oral cavity with pertinent and peculiarly appropriate fitness. In no other subdivision of personal hygiene are the sins of omission fraught with such disastrous and far-reaching consequences as are the disregarding and neglect of the sanitary principles of oral hygiene.

As *mastication* is an important feature of the digestive function, nature has provided suitable organs—the teeth—made not only for service, but also as one of the means of personal adornment when perfectly developed and cared for.

In the practical application of the principles of prophylaxis dental science goes further back in the scale of development than the consideration of the visible or developed teeth. Very much good can be accomplished for the teeth of the child, before its birth, by providing the mother with suitable foods, those which contain the essential elements for the formation of hard tissues, including the teeth, such as lime salts, etc., in the form of lime water (in milk), or the syrup of lacto-phosphate of lime; also certain grains, as wheat, and skim-milk. The latter furnishes a form of lime, the chloride (0.3 to 0.5 percent), which is very essential in building tooth substance. 3

After birth the tooth structure can be improved in the percentage of lime salts by the feeding of cow's

3. "Physical Chemistry," Hammarstein, page 326, third edition.

milk, sterilized, which contains five times as much lime as human milk. 4

Parenthetically, a few words as to heredity may be of interest. Children inherit the individuality and peculiar characteristics of one or both parents, or perhaps of some remote ancestor. They inherit the complexion, the color of the hair and eyes, and the facial contour. They also inherit the peculiarities of the teeth, not only as to size, form, and position in the arch, but the constitutional structure as well.

Each of the four basal temperaments, sanguine, bilious, nervous and lymphatic, are characterized as to form, size, shade, position and texture. 5 When a child inherits a certain temperament it should be endowed with the class of teeth that go with that temperament; though a child may inherit the small teeth of one parent and the massive maxillæ of the other, or the reverse. In the latter case the teeth are crowded and irregular. The correction of such irregularities constitutes a separate department of dental science, termed orthodontia.

. Next after development comes the subject of *sanitation and cleanliness*. The salivary and serumal calculus, which accumulates in some mouths very rapidly, necessitate its removal by a dentist every two or three months. Other mouths may not require this operation in as many years. In the case of young people, in the majority of instances the salivary calculus is the variety usually present. When removed, the surfaces of the teeth should be polished; a slightly roughened surface

---

4. "Physical Chemistry," Hammarstein, page 399, third edition.

5. Lectures upon "Dental Anatomy and Pathology," by Prof. W. C. Barratt.

is favorable, too, and induces future precipitation of the deposit.

The serumal variety—a deposit from the blood—is precipitated in the mouths of persons with a gouty or rheumatic diathesis, 6 some of these cases being benefited by systemic treatment.

After the deposits have been thoroughly removed and the enamel surfaces polished, they may be kept so, by the patient himself, by the diligent and correct use of the tooth-brush and other toilet articles, tooth powders, soaps, washes, picks, silk thread, rubber bands, etc.

The *tooth-brush*—first, as to its selection. In form and size it should be suited to the case. It should be curved to fit the contour of the arch, and the handle also curved, to facilitate minipulation; the bristles firm and well fastened to the handle, with the surface serrated, and an isolated tuft at the end for cleansing the inaccessible surfaces of the teeth, as back of the third molar or in spaces where teeth have been extracted, etc. If the patient be so unfortunate as to wear a "bridge" or other stationary contrivance, then an accessory brush is usually required, a special form and size for these cases.

The correct use of the brush is an important item. To produce the best results, place the bristles against the outer or labial surfaces of the teeth, then brush from the necks of the teeth toward the occlusal surface. Pronate the forearm, or rotate the brush; brush the upper teeth down and the lower ones up (inner or lingual surfaces the same). This will tend to brush the gum festoons into the interdental spaces rather than out

6. Treatise upon "Etiology of Pyorrhea Alveolaris," by Prof. C. N. Pierce, *International Dental Journal*, January, 1894.

of them. The occlusal or masticating surfaces should be brushed from side to side, and forward and backward. This will cleanse the grooves and fissures.

Use the brush as though it were a collection of toothpicks designed to remove the residual food substances which may have become lodged between and around the teeth.

Don't brush across the gums under any circumstances. It will tear them loose from the teeth, unnecessarily bruising and lacerating them. Beware of infection! Do not scour the gums, nor scrub the teeth, nor try to polish them, using the brush as in polishing shoes. Dentists are provided with the proper instruments and materials to perform such operations without injuring either the gums or the enamel of the teeth.

When oily or greasy films and stains accumulate which will not be removed by the brush and luke-warm water, then other agents of detergent character may be brought into requisition. Detergents may be classified into two kinds, chemical and mechanical or physical. Tooth powders belong to the mechanical. They are not of much importance as medicinal applications. The base should be alkaline and should be soluble in the oral fluids, and not too gritty; the grit should be regulated according to the condition of the teeth. Precipitated chalk is good for the basis; the grit can be increased by adding pulverized cuttlefish bone; as a solvent for fats and oils pulverized soap bark or white castile soap is added; and for sweetening use white sugar or saccharine and flavor with some aromatic substance. 14 This general formula can be changed to suit the conditions, but in no case use an insoluble grit such as pumice or charcoal. They work their way under the gingiva or free margins of the gums, establishing a neucleus for the precipitation of calculus and the accumulation of food substances, which latter furnishes a most desirable and comfortable habitat for micro-organisms.

Soaps are chemical detergents which saponify the fats and oils on the teeth, but if used too frequently, or too long continued, will also attack the gums, eventually producing "recession," with consequent exposure of the necks of the teeth.

As a general rule, don't use toothpicks. If you do employ them don't use wooden ones, especially those which are cut—they will splinter; but rather those round, pointed ones, made of hard wood. In the use of picks beware of splinters and infection. Goose-quill toothpicks are the least objectionable 8 when used once or under antiseptic conditions. They are now put on the market in convenient form, with a detachable quill-point in a holder, the latter made telescopic, like a fountain pen, so it may be closed up from dust and dirt (infection).

To properly cleanse the approximal and interdental spaces in some mouths becomes a task. The difficulty

---

14. *Tooth Powder:*

| | | |
|---|---|---|
| R | Precipitated chalk ............ | ℥ 10 |
| | Pulv. cuttlefish bone......... | ℥ 6 |
| | Pulv. sugar ..................... | ℥ 3 |
| | Pulv. castile soap............. | ℥. 1 |

M.—Flavor with oil of rose or the oil of wintergreen, and color with carmine.—N. S. Hoff, D.D.S.

8. "Dental Prophylaxis and Oral Hygiene," lectures by N. S. Hoff.

may be overcome by using silk floss, which is smooth and should be waxed.

By using moderate pressure, accompanied by a sawing movement, a rubber strip will pass between the teeth quite readily and is useful for polishing the approximal surfaces. Some tooth powder may be added to the silk floss or the rubber band to accomplish thorough cleansing.

*Tooth Washes.*15—These are medicinal applications, but may be detergent also. The fundamental principle, however, is that of asepsis. For general use the antiseptic in the formula may be phenol 2 percent or formalin 2 percent. Phenol as in phenol-sodique, formalin in pasteurine and boro-lyptol. Alkalinity may be furnished by the bi-borate of sodium or the benzoate of sodium, the latter reaction being better represented in boro lyptol and benzo-lyptus. The solvent in the tooth wash is alcohol and the diluent is distilled water. Sweeten with saccharine, instead of sugar, as it has antiseptic properties. 8 Flavor with essential oils and color with cochineal.

A mouth wash for general use should not be irritating, but pleasant to the taste. Special conditions

---

15. *Tooth Wash:*

℞ Sacharine.

Soda bi-carb. a a .......... gr. 10

Spirits ........................ ℥ 10

Salicylic acid ................ gr. 10

S.—Ten to fifteen drops in a little water or on a wet brush, after the teeth are cleaned; brush teeth and gums gently. N. S. Hoff, D.D.S.

8. "Dental Prophylaxis and Oral Hygiene," lectures by N. S. Hoff.

require specific mouth washes.[16] A prevalent idea, that all mouth washes should be astringent, is erroneous. Such are required in some cases, but when too frequently used, or too long continued, they will react and produce chronic inflammation of the gums, or an atrophied condition results. Weak alkalies, long used, will produce an atrophied result also.

The best method of application of a mouth wash is by means of a brush. After the teeth are thoroughly cleaned, add ten or fifteen drops to the wet brush and brush the teeth and gums gently. The medicine may be used as a gargle or wash, in suppurative conditions, inflammations, etc.

Green stain is a fungous growth upon the teeth of the mould species. This vegetable garden (it can hardly be classed as ornamental, therefore not a flower bed,) develops and grows in an acid medium. The presence of green stain indicates an acid mucous. This acid has two sources of origin, *fermentation and secretion.* Under certain conditions gums and soft tissues, when irritated and inflamed,[9] or by reason of constitutional or funtional derangement, will secrete a viscid and acid fluid which attacks the tooth, producing *erosion.*

This acidity requires treatment, by the dental physician, both local and systemic. The local treatment

---

16. *Mouth Wash:*

| | | |
|---|---|---|
| R | Thymic acid | ʒ ¾ |
| | Benzoic acid | ʒ 3 |
| | Bi-chlorid | ʒ ¾ |
| | Tinct. eucalyptus | ʒ 15 |
| | Alcohol—absolute | ℥ 12½ |
| | Oil wintergreen | min. 25 |

M. S.—Fifteen to 20 drops in one-third tumbler of water. PROF. MILLER.

9. "Dental Caries," by Prof. G. V. Black, *Journal of Am. Med. Assn.*, vol. 4, p. 85.

consists of a process of neutralization by an alkali, best accomplished by Phillip's milk of magnesia. This magnesium hydrate is not only alkaline, but it is also antiseptic. In a strength of 1 to 2000 it is antiseptic or prevents the growth of the strepto coccus pyogenes. 10

Functional derangements of the oral tissues and membranes, such as catarrhal inflammations or reflex irritations from digestive disturbances, also those produced by eructations from the stomach, require special additional treatment.

*Bacteria and Fungi.*—The part played by bacteria and fungi in the pathology of the mouth is an interesting topic. The effects produced may be classified into two groups, those which affect the soft tissues and those which disease the hard structures, the teeth. Of the affections of soft tissue may be mentioned thrush, a membranous disease produced by a mould.

Yeast produces fermentations, and is now being accused of producing cancer. This question is still under investigation.

Bacillus tuberculosis is actively represented in lupus, a mouth disease, being a variety of skin tuberculosis. Of the other pathogenic bacteria we might mention those of bacillus diphtheria, pneumonia croupos, Franckel's diplo coccus, strepto coccus pyogenes, and a number of vibriones, bacilli and spirochaetes which can not be grown upon artificial media. 11

Of the acids produced in the mouth we have lactic, butyric, acetic, etc. Of the pus micro-organisms, there are myriads of them.

---

10. Exp. in Bact. Laboratory, University of Michigan, by B. L. Kesler, D.D.S., 1901.

11. "Bacteria in the Pulp, by Prof. Miller, Berlin; *Dental Cosmos*, 1894.

Whenever the tissues become less resistant or below normal, through impaired nutrition, wounds or traumatic lesion, then the invasion begins the campaign being waged so persistently and vigorously that septicamia pyemia and death often result. 11

Of the purulent conditions the most serious ones are antral abcess and dentigerous cysts; of abcesses of the oral cavity we have the whole family, from the superficial gum boil to the deep seated alveolar abscess; abscesses which see with one or more eyes (fistula), and those which do not see, in fact totally "blind"; those which are hot, feverish and acute, and those which are "cold," indolent and slumbering. Special treatment is indicated for each condition.

*The Teeth.*—The greatest havoc wrought in the mouth by bacteria, or the condition most keenly realized by the patient, perhaps, is that of caries, or decay of the teeth.

The process of decay, briefly stated, is as follows: The starches of the food residuum are converted by ptyalin into maltose; maltose undergoing fermentation by a bacillus, produces lactic acid; the acid dissolves the lime salts of the enamel and dentin, the organic matrix remaining becoming liquified or decomposed by other bacteria, those producing soluble ferments 9 in either acid or alkaline media, the by-product then becoming food for saprophitic or other putrefactive micro-organisms. Each species in turn performs a certain and definite work, and is finally killed by its own toxin or product. This product, which was a poison to

---

11. "Bacteria in the Pulp," by Prof. Miller, Berlin; *Dental Cosmos*, 1894.

9. "Dental Caries," by Prof. G. V. Black, *Journal of Am. Med. Assn.*, vol. 4, p. 85.

the former tenant, now becomes food for the subsequent occupant. The process continues till complete disorganization of the complex molecule is accomplished.

An important fact to be remembered is, that the very first step in the process of decay is the production of acid; and in the majority of instances that acid is a product of bacterial fermentation as a result of the uncleanliness of the teeth.

What shall we do for that bad breath? Remove the cause which is producing it. Temporarily, as an expedient, a deodorant will either mask or destroy the odor. In the latter case the gas is made to form a chemical compound by uniting with some other chemical substance. 13 The fetid breath is caused by derangement of the stomach and intestinal organs, or it may be a putrid condition of the oral cavity. 12 For proper treatment consult a dentist.

As may be inferred from the outline given of some of the operations and treatments to be performed by an oral physician or surgeon, the dentist of the future must know more than to merely extract a tooth and make a plate, or to insert a gold filling and build a bridge.

The dentist should know enough about the sciences of bacteriology and pathology to realize the importance of asepsis in all operations upon the oral tissues; not merely mechanical cleanliness, but surgical and bacterial also. The latter training can only be acquired in a well-regulated bacteriological laboratory.

A few suggestions might be offered to aid you in the selection of a dentist. First of all the dentist should

---

13. Bacteriology Lectures, Prof. Novy, University of Michigan, Dec. 10, 1900.

12. "*Fetor ex ore*," "Deposits on the Teeth," by Prof. Miller, Berlin, *Dental Cosmos*, vol. 36.

be honest, conscientious and thoroughly reliable, a person in whom you can place implicit confidence ; and secondly, he should be thoroughly trained in dental science, which can only be accomplished in a reputable college, *competency* and *qualification* therefore being assured.

